# The outcome of the forensic triage preceding disaster victim identification in the downing of Malaysia Airlines flight 17

**DOI:** 10.1080/20961790.2022.2043611

**Published:** 2022-11-04

**Authors:** Erwin Vermeij, Peter Zoon, Reza Gerretsen, Vincent Otieno-Alego

**Affiliations:** a Microtraces and Materials, Netherlands Forensic Institute, Den Haag, The Netherlands; b Forensic Anthropology and Archaeology, Netherlands Forensic Institute, Den Haag, The Netherlands; c Operational Science and Technology, Australian Federal Police, Canberra, Australia

**Keywords:** Forensic triage, DVI, air crash, fragments, surface to air missile, warhead

## Abstract

Malaysia Airlines flight 17 crashed on 17 July 2014 while flying over an area of armed conflict in eastern Ukraine. The first forensic trace evidence was collected after the human remains were transferred to a safe location in the Netherlands for identification and repatriation. Disaster victim identification processes were therefore undertaken in concert with the forensic investigation. Prior to these processes, X-ray and computed tomography scanners were used to reveal foreign objects in the human remains, and a large number of these fragments were recovered after the forensic triage. A distinct group of metal fragments was identified as being potential remnants of high-energy foreign objects. Forensic analysis revealed that they were explosively deformed unalloyed steel fragments, some of which had shapes consistent with pre-formed metal fragments found in a 9N314M warhead used in Buk SA-11 missiles. Furthermore, thin film deposits of cockpit glass and aluminium were identified on the most heavily deformed side of some of the explosively deformed metal fragments, suggesting they came from outside the airplane. These findings supported early suspicions that Malaysia Airlines flight 17 was struck by a Buk SA-11 missile.

**Keypoints**
- A multidisciplinary approach for combined identification and forensic investigation of human remains after a mass fatality incident.
- The combined use of complementary X-ray techniques for detection and provisional characterization of foreign objects in human remains.
- The use of sensitive and highly discriminative state of the art techniques for analysing foreign objects recovered from human remains.

## Introduction

Malaysia Airlines flight 17 (MH17) was a scheduled passenger flight from Amsterdam to Kuala Lumpur. On 17 July 2014, MH17 crashed in an area of armed conflict in eastern Ukraine, killing all 283 passengers and 15 crew members. Contact with the Boeing 777-200ER aircraft was lost when it was approximately 50 km from the Ukraine–Russia border. This was Malaysia Airlines’ second aircraft loss in 2014 after the disappearance of Flight 370 on 8 March. Wreckage of the MH17 aircraft was spread across a 50 km^2^ area southwest of Hrabove, Donetsk Oblast. Hours after the crash, news reports showed images of wreckage containing multiple puncture holes and indentations.

According to the International Civil Aviation Organization, the country in which an aviation incident occurs is responsible for the investigation into its cause, but that country may delegate the investigation to another state [1]. In this case, Ukraine delegated responsibility to the Netherlands, who led two separate investigations. The Dutch Safety Board (DSB) conducted an investigation into the technical cause of the crash and released their report on 13 October 2015 [2]. In parallel, a criminal investigation was conducted by a separate joint investigation team (JIT), which consisted of police officers and public prosecutors from Australia, Belgium, Malaysia, the Netherlands and Ukraine and was coordinated by the public prosecutor of the Netherlands.

To gather technical evidence from a crash site, investigators normally have the opportunity to visit the site and examine the debris *in situ*. Materials and traces of relevance are then collected for further examination. In the case of MH17, two main problems existed. First, the crash site was in an area of armed conflict, making it unsafe for investigators to view the wreckage *in situ*. Second, the site was unguarded and therefore open to evidence tampering.

In the days following the crash, it was unclear if and when the site would be accessible to forensic investigators. The first and at that time the only opportunity to look for evidence was during the identification of human remains. Therefore, the identification process was considered part of the criminal investigation. Prior to identification, the human remains were searched for traces that might be of forensic importance.

The first coffins containing human remains were flown to the Netherlands on 23 July 2014. The identification of passengers and crew took place at the Korporaal van Oudheusden barracks in Hilversum and was coordinated by the National Forensic Investigation Team of the Netherlands. On 26 July, the first victim had been identified.

By 5 December 2014, the Dutch-led disaster victim identification (DVI) team had identified 292 of the 298 passengers and crew. Later searches of the crash site led to the identification of four more passengers by April 2015. Sadly, the remains of two Dutch citizens on the flight have not been recovered.

## The forensic triage

There were two main objectives for the teams working in Hilversum: (i) forensic investigation and (ii) victim identification. In the Korporaal van Oudheusden barracks in Hilversum, a forensic triage was used to sort remains for direct identification or identification after a forensic investigation. A forensic triage is a process for collecting, sorting and prioritising evidence of a crime when limited resources must be allocated. At the start of the triage, it was unclear what had caused MH17 to crash. Different scenarios were proposed, including: (i) an accident due to technical problems, (ii) an explosion inside the airplane caused by an improvised explosive device, (iii) an explosion outside the airplane caused by a missile and (iv) damage caused by 30 mm rounds or air-to-air missiles fired by a Sukhoi SU-25 jet.

Identification of human remains began in Donetsk the day after the crash. The local pathologist took photographs, wrote descriptions, took bone samples from 12 bodies for DNA testing. Subsequently, the decision was made to continue the identification process in the Netherlands, and the human remains and DNA samples were transferred there [3]. The identification process was then carried out by 120 forensic specialists from the National Forensic Investigation Team of the Netherlands and 80 forensic specialists from Australia, Belgium, Germany, United Kingdom, Indonesia, Malaysia and New Zealand [4, 5].

Prior to identification, selected human remains underwent a “forensic investigation” involving a search for traces of forensic relevance in a manner designed to avoid impacting the prioritised DVI. In addition, autopsies were carried out on a limited number of bodies. The forensic investigation and autopsies were carried out by staff of the Netherlands Forensic Institute (NFI).

### Description of forensic operations

**Figure 1.**
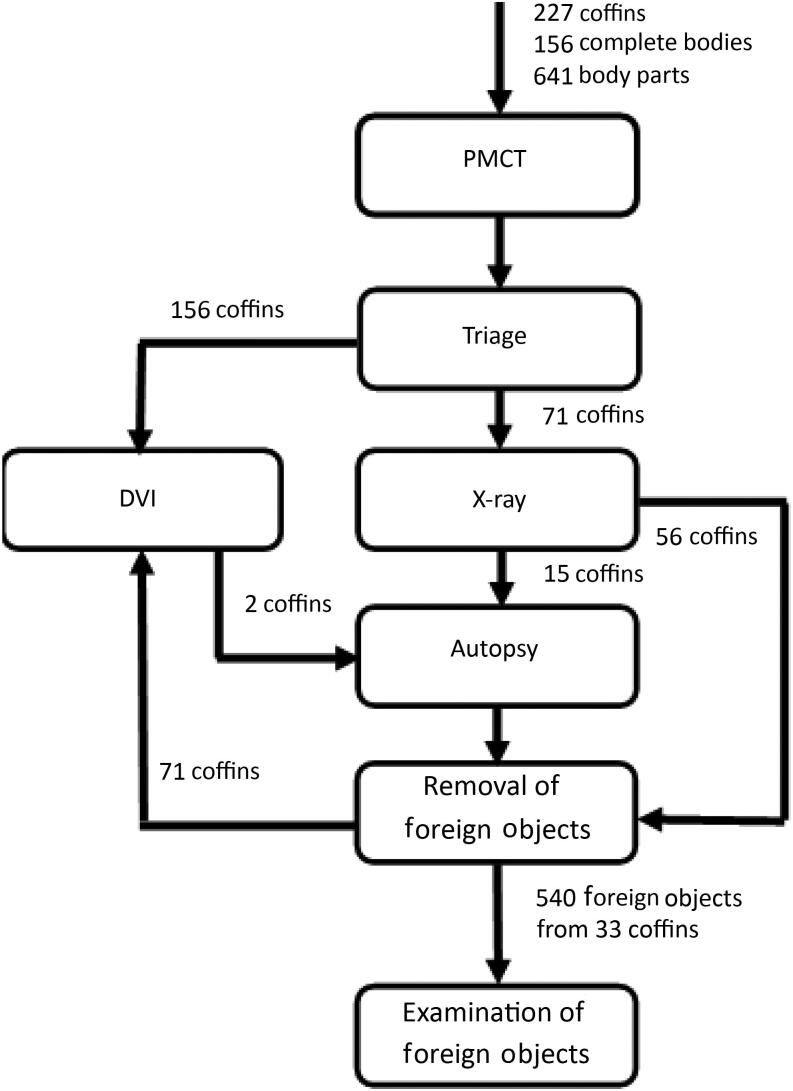
Schematic presentation of the forensic triage conducted at the Korporaal van Oudheusden barracks in Hilversum, the Netherlands. To assist the triage, an additional “street” equipped with on-site computed tomography (CT) and X-ray equipment was set up for the forensic investigation. PMCT: post-mortem CT. DVI: disaster victim identification.

To facilitate the detection and recovery of foreign objects with minimal disruption to the DVI, an additional “forensic process” line equipped with computed tomography (CT) and X-ray scanners was introduced. After the coffins arrived in Hilversum, they were placed in cold storage containers. At the start of the process, the contents of each coffin were screened by military staff for chemical and biological hazards. Because of the formaldehyde treatment of remains in Ukraine, all staff wore gas masks. After the safety screening, post-mortem CT (PMCT) scans of the body bags from the coffins were made, following the process presented in Figure 1. A large number of the PMCT scans revealed foreign objects both in and on the human remains in the body bags. After the remains were visually examined, some underwent an additional X-ray scan while others were directly forwarded for identification. Remains showing foreign objects were diverted for further examination and object removal, then released for identification.

During the triage, 227 coffins were processed, of which 156 were selected for direct identification and 71 underwent forensic examination [4]. From approximately 33 human remains, approximately 540 objects without a readily identifiable source were secured and sent to the NFI for further examination. Three crew members were autopsied during the forensic examination in Hilversum and two others underwent an autopsy at the NFI after identification.

### Detection of foreign objects

Foreign objects in the human remains were detected using a medical CT scanner and a customs dual-energy X-ray scanner. An X-ray beam undergoes an attenuation that depends on the density and thickness of the object through which it passes. CT reconstructs three-dimensional (3 D) images from many two-dimensional images acquired by directing X-rays through an object at different angles. Industrial CT scanners rotate the object in the X-ray beam, whereas medical scanners rotate the X-ray tube around the body [6].

PMCT has been used several times in recent DVI processes and become an integral part of autopsy practice [7–11]. In a forensic context, PMCT is mainly used for identification of factors contributing to the cause and manner of death, DVI and the detection, localisation and identification of foreign materials and objects in human remains [7]. Metallic objects within remains may lead to artefacts that restrict analysis of surrounding tissues. To minimise artefacts and improve material characterisation, dual-energy CT may be used [12–14]. However, this method cannot normally be used to determine the composition of materials and objects [15]. Because most foreign objects encountered in a forensic investigation (e.g. bullets, shrapnel) are composed of dense metals (e.g. steel, brass, lead, tungsten), their discrimination requires X-ray energies of 200–300 kVp. To avoid tissue radiation damage, the X-ray tube voltage in medical CT scanners is limited to 150 kVp. Industrial scanners may use energies up to 320 kVp [16], but these scanners require the body to be rotated, which makes them unsuitable for PMCT. Moreover, higher X-ray energies may also affect or hamper autopsy findings [15]. In Hilversum, a mobile 16-slice clinical scanner operating at a fixed tube voltage of 120 kVp was used.

Baggage and cargo X-ray scanners are used for scanning luggage and cargo for customs and security purposes. Similar to medical X-ray scanners, an X-ray beam undergoes attenuation dependent on the density and thickness of the object in the direction of the beam. Baggage and cargo scanners mechanically pass the object through the beam, and the transmitted X-rays are detected using a linear array, which builds up an image that can be seen by the operator.

In a dual-energy system, the detector is constructed in two layers to distinguish low- and high-energy X-rays [17]. Compared with a single-energy system, the major advantage of a dual-energy system is its ability to differentiate materials according to their atomic number by comparing attenuation ratios of low- and high-energy X-rays. This technique provides the operator with colour-coded images in which the colour indicates the chemical composition of the scanned object. The colour depends on the atomic effective number (Z_eff_) of the material at each pixel location and is recognised as organic, inorganic or metallic. Most manufacturers use shades of orange to identify organic material (Z_eff_ < 11) and shades of blue or purple to identify heavier elements (Z_eff_ > 18) such as iron, steel, gold and silver. In the intermediate range (11 < Z_eff_ < 18), shades of green identify materials such as glass, aluminium and some plastics, while materials that are impenetrable by X-rays appear black.

During the triage, a self-made calibration standard was placed on top of the coffins and scanned simultaneously with the remains. The standard consisted of a Perspex strip embedded with 3-mm thick pieces of stone, aluminium alloys (two types), stainless steel, brass, copper, tungsten and lead. To reduce the number of objects detected, the standard settings of the scanner were adjusted so that aluminium, glass and bone fragments appeared orange and metals heavier than aluminium appeared green or blue. In Hilversum, a customs dual-energy system was operated at a tube voltage of 160 kVp.

### Removal of foreign objects

The CT scans identified foreign objects in a large number of the bodies of passengers and crew members. After X-ray scanning, 15 bodies underwent a full or limited autopsy and 56 underwent external and internal examinations to remove foreign objects. After identification, two further autopsies were performed at the NFI, one on the remains of a relief flight crew member and another on a cabin crew member.

Upon removal of the foreign objects, a distinction was made between material found on the remains and that found within. Distinctions were also made between material found under and within the skin and between magnetic and non-magnetic materials. Objects with no obvious connection to the incident (e.g. coins, zippers, watches) were not removed for forensic purposes. Approximately 540 foreign objects with different origins were removed from approximately 33 human remains and sent to the NFI for further examination.

Most foreign objects were detected in the remains of two flight crew members and a cabin crew member, all of whom were concluded to be in the cockpit at the time of the crash. In the remains of one flight crew member (flight crew member #1), about 200 foreign objects were detected. In the remains of the other flight crew member (flight crew member #2) and the cabin crew member, more than 100 foreign objects (mostly metal) were detected. During autopsy, approximately 220 foreign objects were removed from these three bodies for forensic examination. Based on the autopsies, it was concluded that the three crew members sustained multiple fatal injuries associated with the high-velocity impact of metal fragments.

Relatively few foreign objects were detected in the remains of the other 295 passengers and crew. The passengers and other crew members suffered multiple fractures consistent with in-flight disintegration of the airplane and ground impact. The centre of the airplane was severely damaged and burnt. Consequently, most human remains from this section were fragmented and partly digested by fire. Fragmentation, fire and possibly decomposition and animal scavenging explain why few or no human remains were found for some of the passengers.

### Examination of foreign objects

When an explosive charge is detonated, the resulting shockwave causes fragmentation and very severe damage to all materials next to the seat of the explosion. Fragments are expelled at very high speeds by the hot and rapidly expanding gases resulting from the explosion. The main signatures of an explosion are sooting, heat scorching, melting, penetration, cratering (pitting) and the formation of heavily deformed fragments bearing characteristic morphological features [18].

Fragments removed from the human remains were first screened for explosive residues using an ion-mobility spectrometer. They were then washed sequentially with methanol and ultrapure water to extract the organic and inorganic explosive residues, respectively. The results of the explosive residue analysis were not the subject of this study and are therefore not discussed further. After the explosive residue extraction, remnants of human tissue on the fragments were removed by washing in an aqueous enzyme solution. The cleaned fragments were then air-dried and photographed using a low-power microscope. Preliminary examination of the fragments involved measuring their weights and sizes, recording their magnetic properties and inspecting their surfaces for markings, paint and signatures of an explosion. Sooting was not a very discriminative signature because a large part of the airplane had been on fire.

To reveal morphological features and elemental compositions, a selected number of foreign objects were investigated using light microscopy and either a Quanta 400 ESEM (FEI Company, Hillsboro, OR, USA) or Quanta 3 D FEG 600 dual beam (FEI Company) scanning electron microscope (SEM). A dual beam SEM incorporates both a SEM and a focused ion beam (FIB) in a single system. The SEM accelerating voltage was 15–25 kV, depending on the particle characteristics and analytical goals. For chemical analysis, both SEMs were equipped with an energy-dispersive X-ray microanalysis system (EDS) comprising a silicon drift detector (Oxford Instruments, Abingdon, Oxfordshire, UK) and AZTEC or INCA software (Oxford Instruments). SEM-EDS analyses were typically performed in the low vacuum (10–20 Pa) mode of the SEM. The focused ion beam FIB of the Quanta 3DFEG 600 microscope was used to sputter material from the surface and make cross-sectional cuts in the surface of some fragments. The electrons induced by the ion beam were used to create an image of the cross sectional cuts.

The elemental composition of some foreign objects was analysed in greater detail using laser ablation inductively coupled plasma mass spectrometry (LA-ICPMS). This is an highly discriminative analytical technique by which very small quantities of material are ablated and brought into a gas stream as an aerosol. In an argon plasma the aerosol particles are evaporated, atomised and ionised. Subsequently, the ions are identified and quantified with a mass spectrometer.

## Results and discussion

Anti-aircraft weaponry and improvised explosive devices packed with bolts or nails spread shrapnel and heavy objects towards their target. Therefore, the forensic investigation focused on heavier materials such as steel, tungsten and lead.

**Figure 2.**
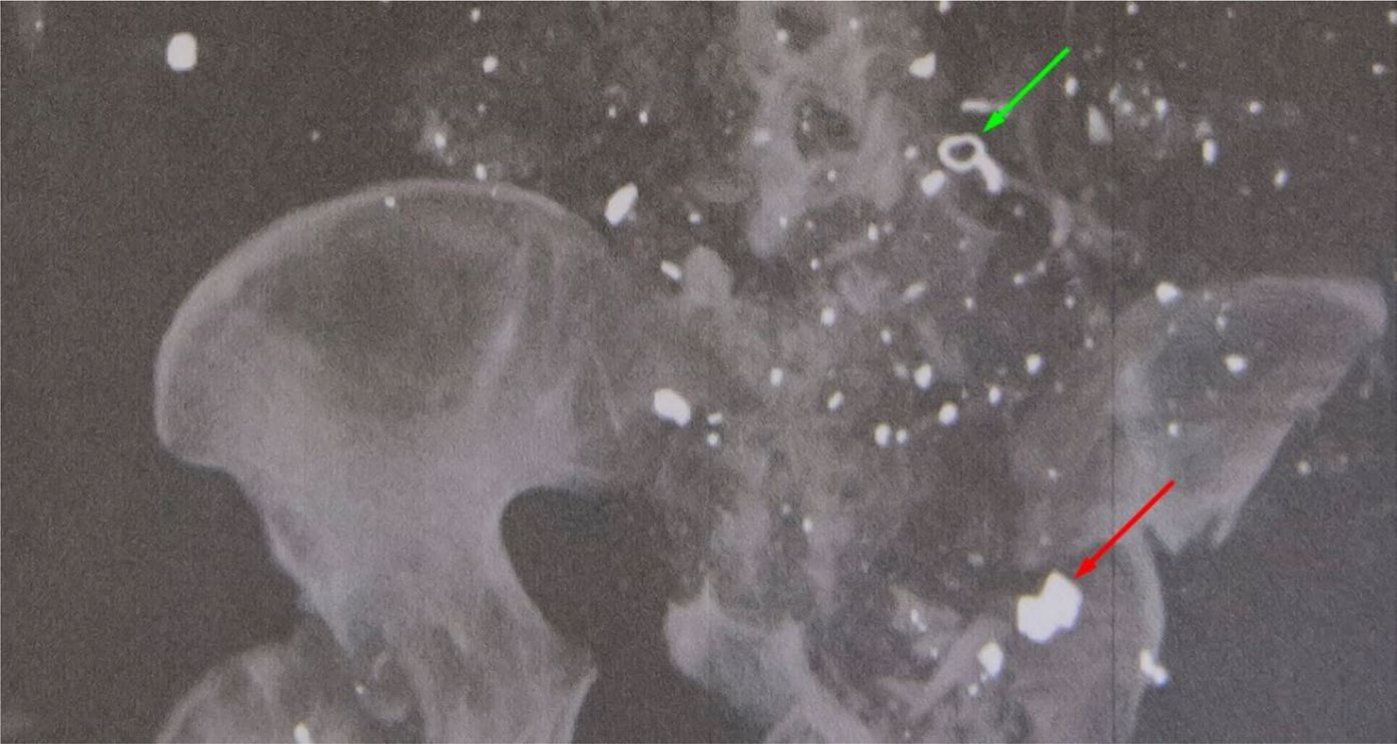
CT topogram of the remains of flight crew member #1, showing a large number of foreign objects, including a ring-shaped (green arrow) and bowtie-shaped (red arrow) fragment.

**Figure 3.**
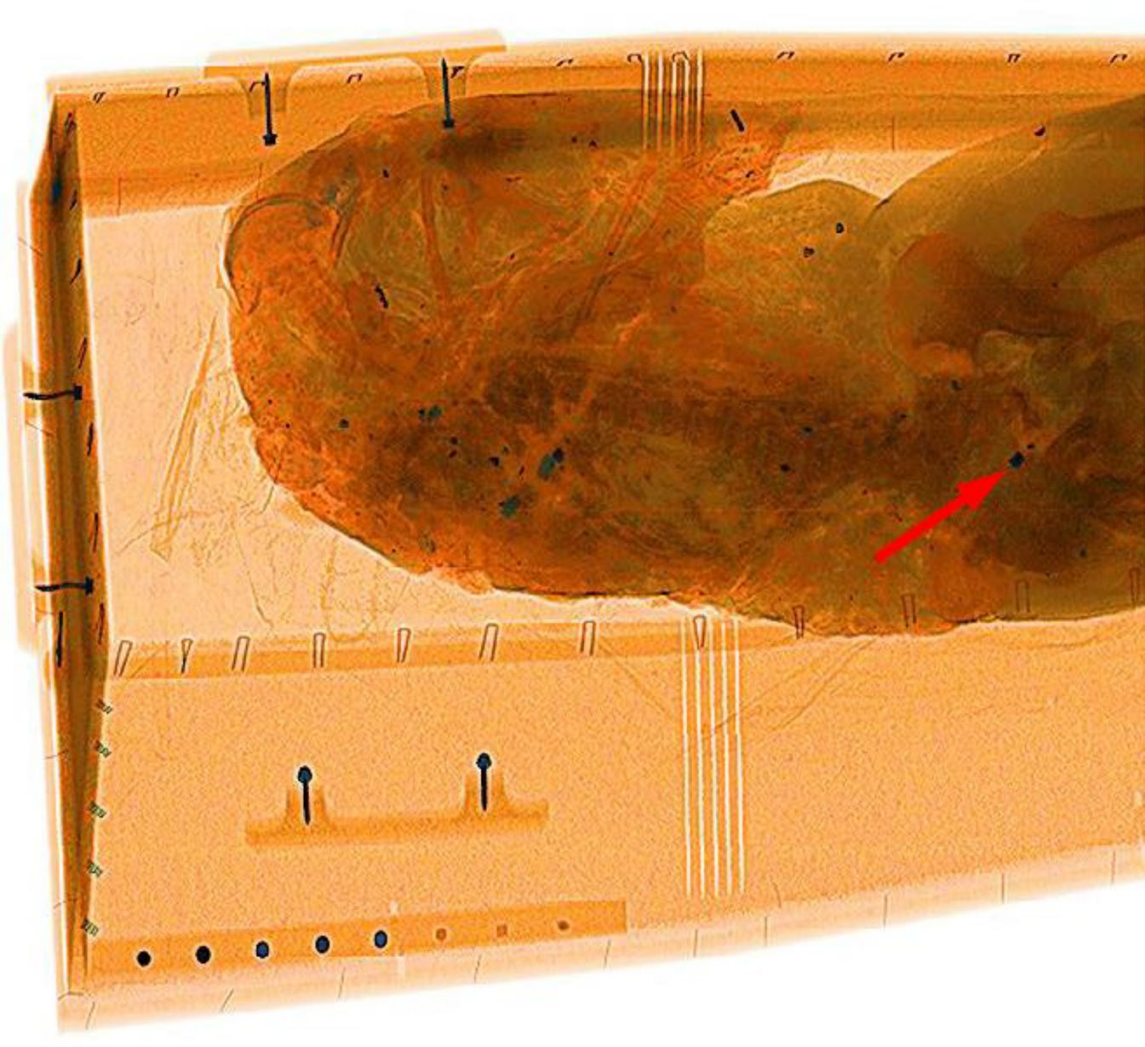
X-ray image of the remains of flight crew member #1. The self-made calibration standard (lower left) was used to determine the probable composition of the foreign materials in the remains. The red arrow indicates the bowtie-shaped fragment identified in Figure 2.

A CT topogram and X-ray image from flight crew member #1 are shown in Figures 2 and 3, respectively, and indicate that the remains contained many foreign objects. In the CT image, these materials appear mostly white or light grey, whereas they appear dark in the X-ray image. Although the two-dimensional topogram provides limited information about the type of material, it is based on 3 D imaging data that provides information about the relative positions of foreign objects in the body.

**Figure 4.**
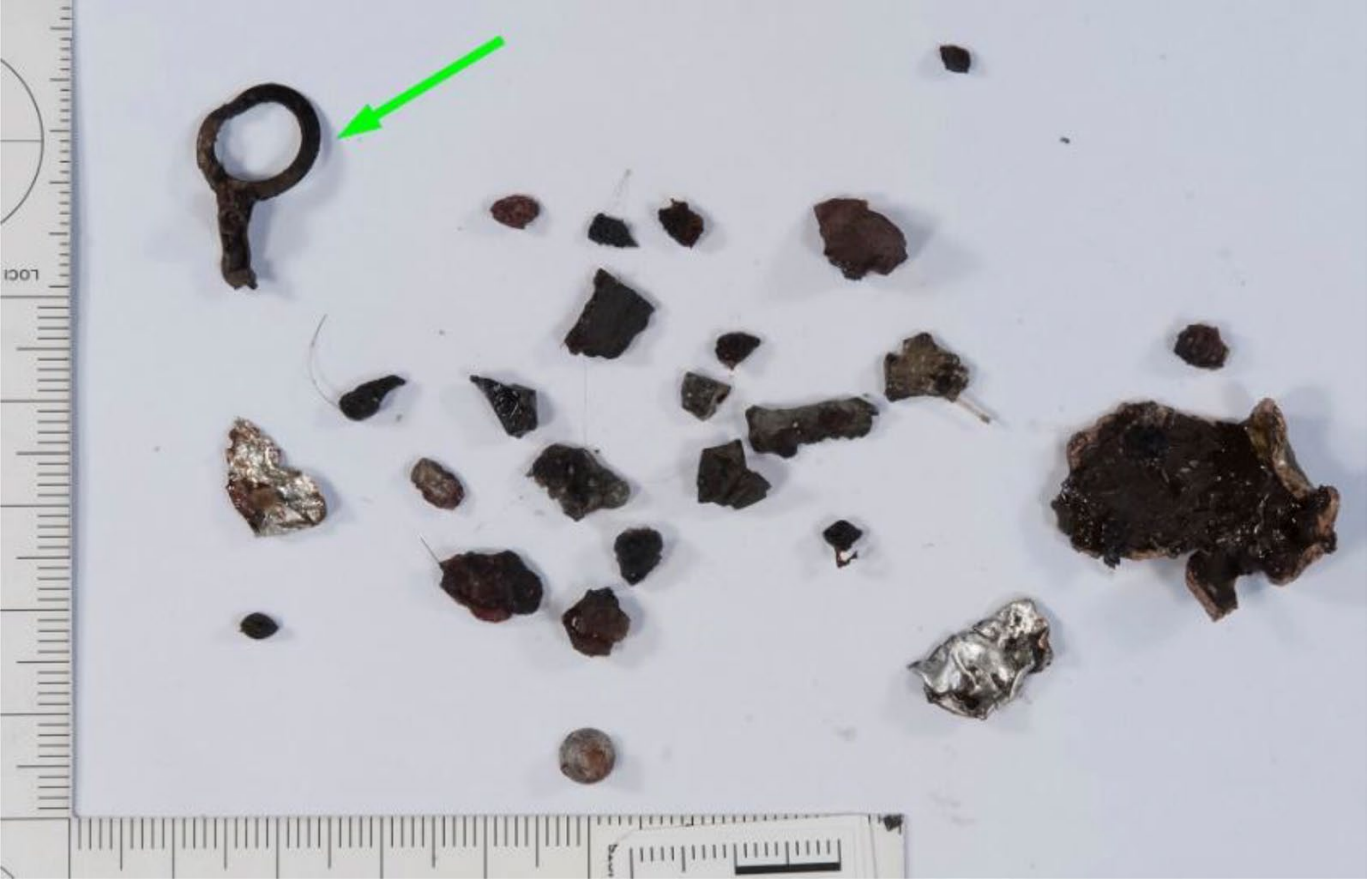
Photograph showing some of the foreign objects recovered from the remains of flight crew member #1 before cleaning. The ring-shaped object (green arrow) is also visible in the CT topogram (Figure 2). Scale: mm.

Some of the foreign objects recovered from the remains of flight crew member #1 are shown in Figure 4 and include a distinctive ring-shaped object clearly visible on the topogram in Figure 2 (green arrow). Notably, the ring-shaped object is not visible in the X-ray image in Figure 3 because it is made of bronze coloured aluminium and the scanner settings were adjusted (using the self-made standard) such that aluminium, glass and bone fragments all appeared orange. This approach reduced the clutter compared with that observed in the topogram and allowed easier localisation of the heavier metal-like particles, which appeared green or blue.

**Figure 5.**
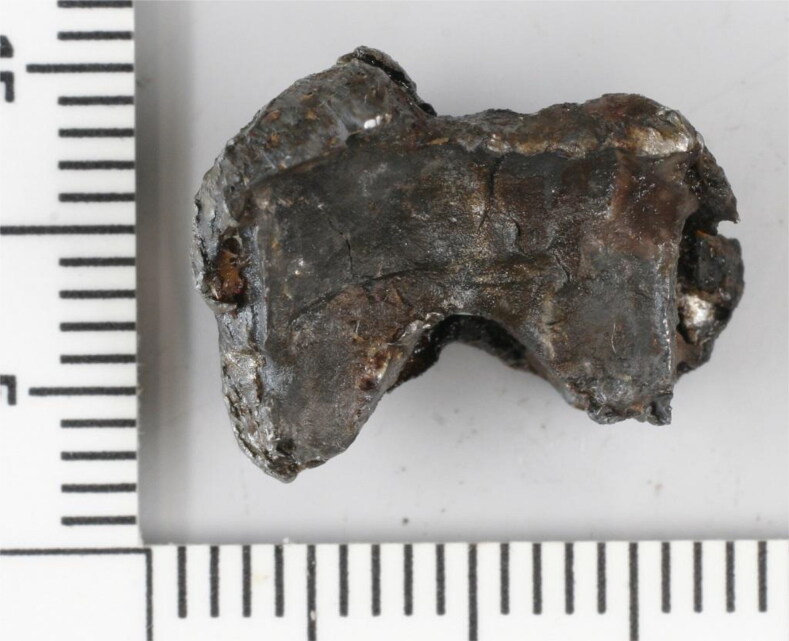
Photograph of a bowtie-shaped steel fragment recovered from the remains of flight crew member #1. Scale: mm.

**Figure 6.**
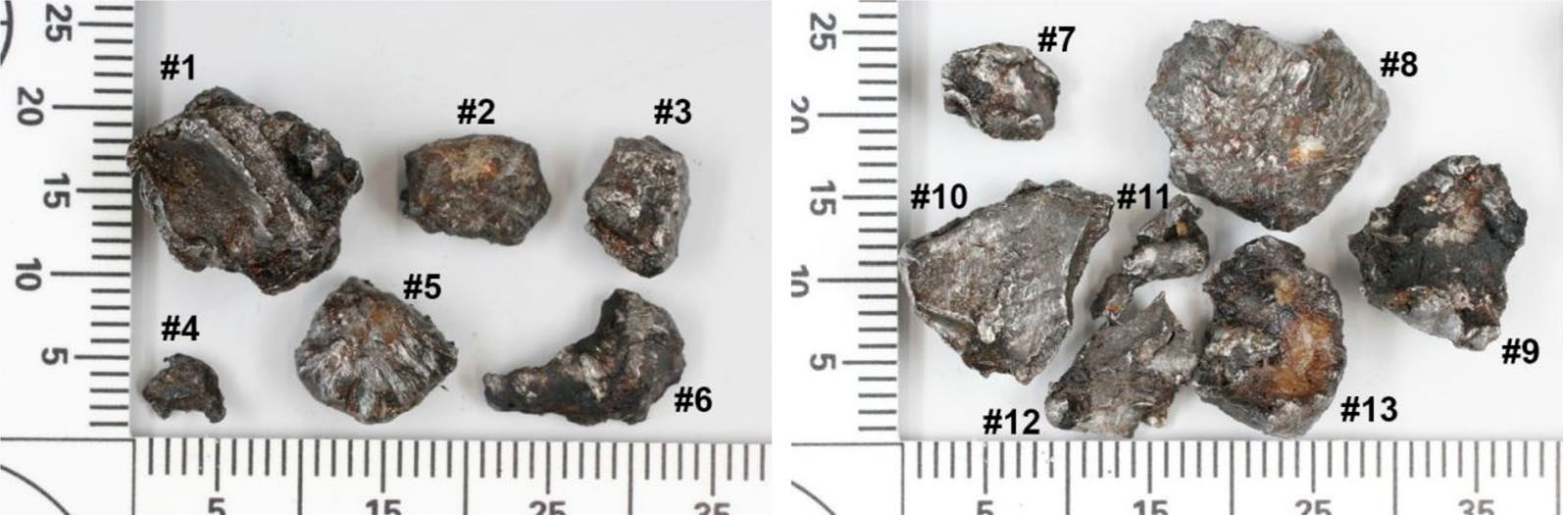
Photographs of 13 steel fragments recovered from the remains of flight crew member #2. Scale: mm.

One of the fragments secured from flight crew member #1 had a distinctive bowtie or butterfly shape (Figure 5). This fragment is clearly visible in the topogram in Figure 2 and X-ray image in Figure 3 (red arrows). The fragment consisted of an unalloyed steel, similar to 13 other heavily deformed fragments found in the remains of flight crew member #2 (Figure 6). The identification of these 13 fragments and the bowtie-shaped fragment suggested that steel was a target material for recovery. Thirty steel fragments were recovered from the three crew members, some with a distinct shape.

**Figure 7.**
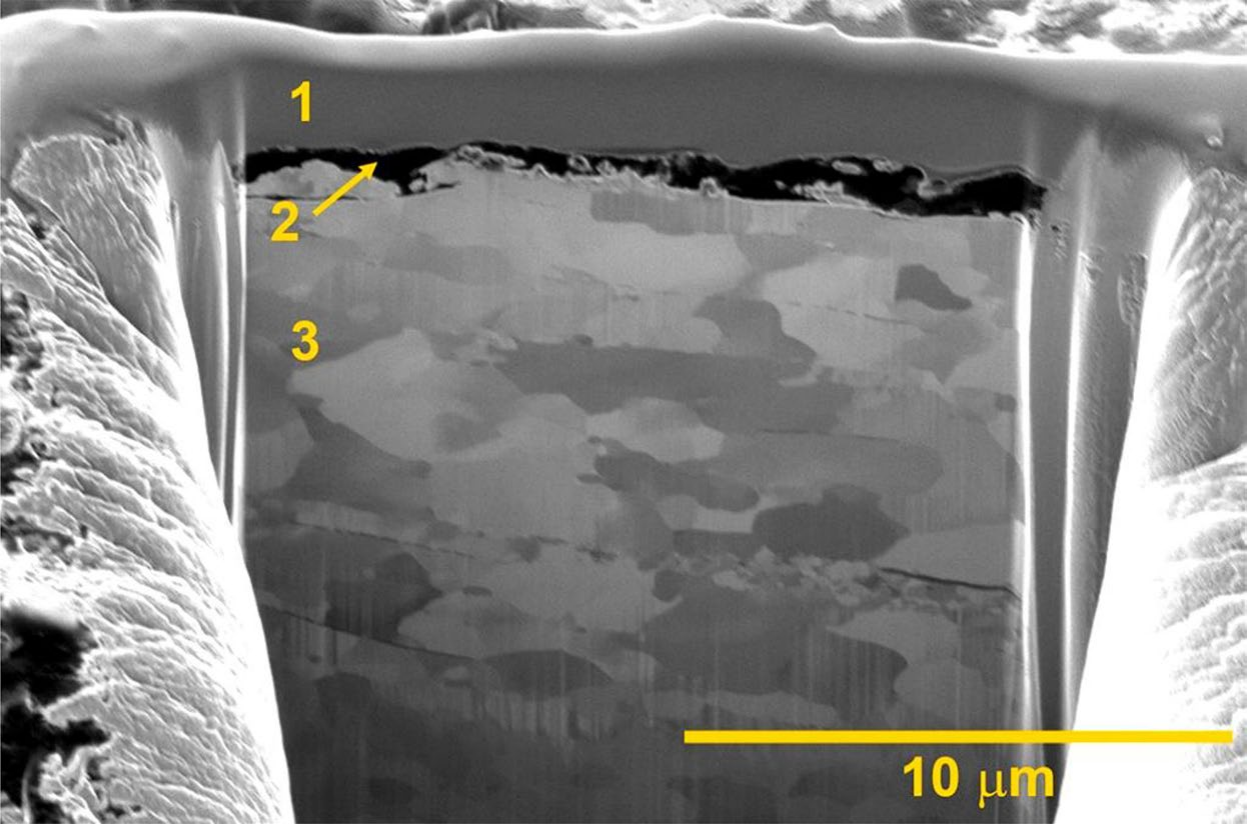
Focused ion bean (FIB) secondary electron image showing a cross-section of fragment #8 (Figure 6) recovered from the remains of flight crew member #2. Prior to imaging, the surface layers were exposed using an FIB. The elemental compositions of the layers were analysed using energy-dispersive X-ray microanalysis. 1) Protective layer of platinum deposited by the Netherlands Forensic Institute (NFI). 2) Layer of re-solidified cockpit glass. 3) Underlying steel.

In early August 2014, a FIB was used to expose surface layers of some deformed steel fragments. A representative cross-section of one of the fragments recovered from the remains of flight crew member #2 is shown is Figure 7. SEM-EDS analyses of these cross-sections identified surface layers of resolidified aluminium and glass, with thicknesses ranging from a few micrometres to tens of micrometres. The surface layer of glass on selected fragments had an elemental composition comparable with that of exemplar cockpit window glass, which contains a relatively large amount of zirconium (≈2%–4%) that is not present in common types of glass, such as that used for ordinary windows.

Further examination of the bowtie-shaped fragment showed it was heavily deformed on one side. Deposits of glass and aluminium were mainly found in a re-solidified state on the deformed side of the fragments, indicating that the fragments impacted the airplane at a very high velocity. The consequential frictional heat melted the glass and aluminium and a thin, solidified layer of these materials was deposited on the heavily deformed side of the fragments. Although their velocity was reduced after impacting the airplane, these fragments continued on their trajectory and impacted the crew members, wherein they were found.

In December 2014, the first pieces of wreckage arrived in the Netherlands. During the examination of the wreckage, more steel fragments were recovered. LA-ICPMS was used to compare the chemical composition of selected steel fragments recovered from the wreckage with that of fragments recovered from the human remains. Principal component analysis of these data showed that the fragments clustered into groups with similar chemical composition. Notably, the bowtie fragment recovered from the remains of flight crew member #1 (Figure 5) appeared in the same group as a bowtie-shaped fragment later recovered (in January 2015) from cockpit debris during the wreckage examination. This suggests that both fragments came from the same source (e.g. a warhead).

**Figure 8.**
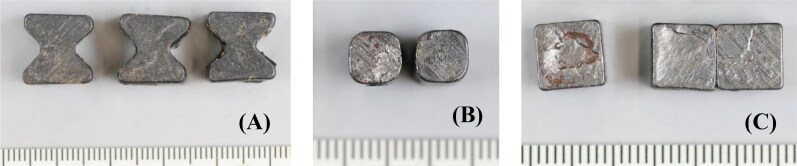
Photographs of representative 9N314M warhead fragments. (A) Bowties. (B) Fillers. (C) Squares. Scale: mm.

The bowtie-shaped metallic fragment recovered from the body of flight crew member #1 (Figure 5) was characteristic of the pre-formed fragments contained in six 9N314M warheads that were dismantled to support the JIT investigation. The 9N314M warhead is carried on the 9M38 series of Buk SA-11 surface-to-air-missiles. The pre-formed fragments from a 9N314M warhead have three distinct shapes: bowties, fillers and squares (Figure 8). These fragments are made of low-carbon hot-rolled ferritic steel with a Vickers hardness of approximately 200 kgf/mm^2^. The gross chemical composition of the fragments recovered from the flight crew and cockpit could not be differentiated from that of the pre-formed fragments from the dismantled warheads.

**Figure 9.**
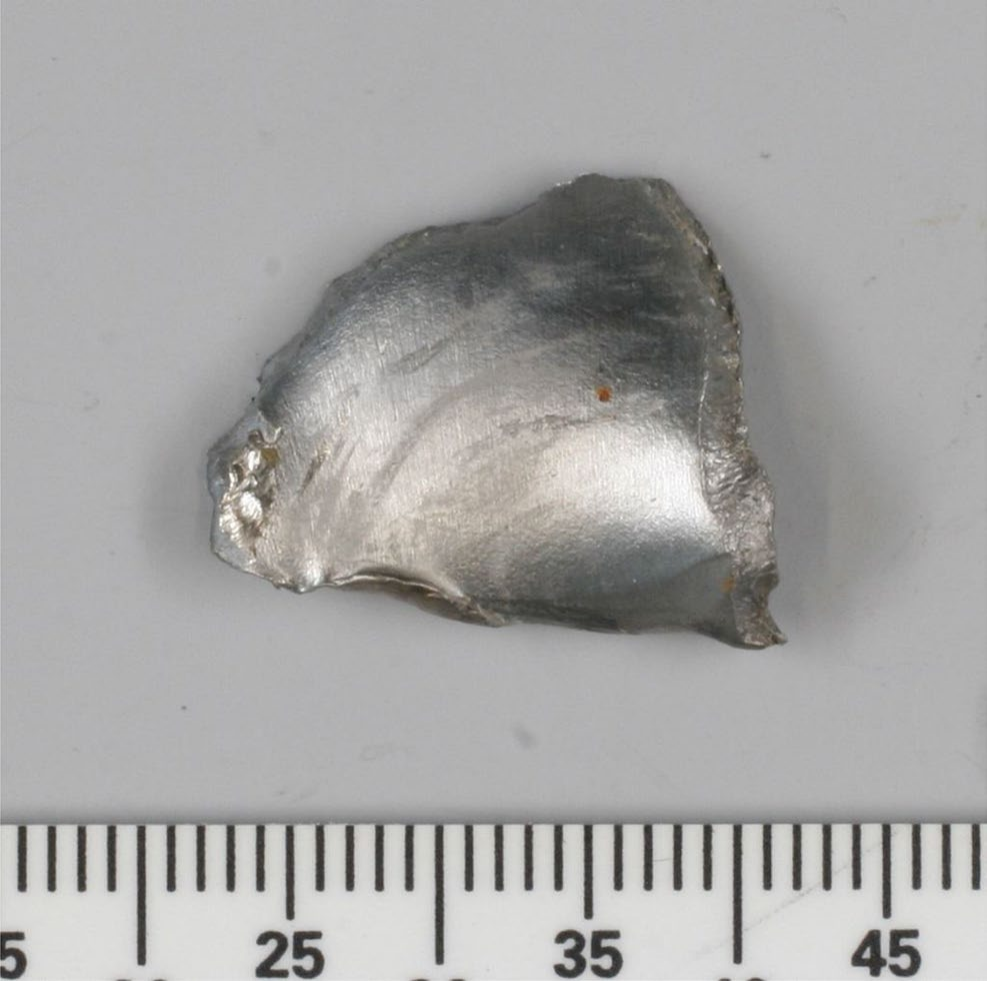
Photograph of a stainless steel fragment recovered from the remains of a relief flight crew member. Scale: mm.

**Figure 10.**
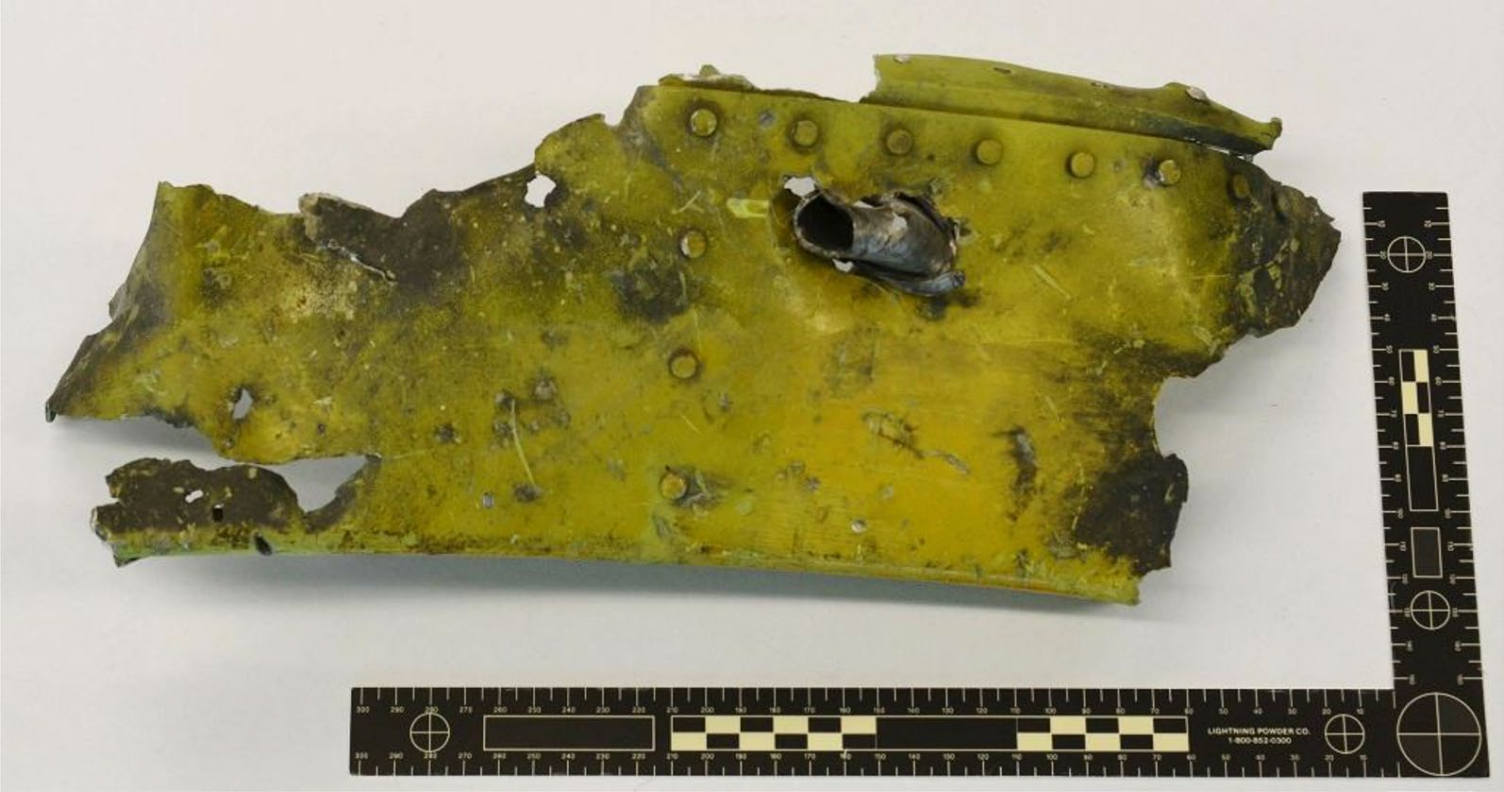
Photograph showing a piece of airframe pierced with a metal fragment.

Another important fragment was recovered from the remains of one of the two relief flight crew members, although its importance was not recognised until late 2014. The stainless steel fragment depicted in Figure 9 was secured on 31 July 2014. Two weeks later, a piece of the airframe, pierced with a metal fragment, was submitted for examination (Figure 10) and found to bear unique numbers that made it possible to identify its original location on the fuselage. The piece of airframe was identified as part of a cross beam located slightly behind and approximately in line with the top of the rear left cockpit window, just behind the cockpit and just above the crew rest area. The embedded metal fragment was not removed from the piece of airframe until late 2014, and its relationship to the fragment recovered from the relief flight crew member was not established until shortly thereafter. The chemical composition and striations on each side of both fragments were consistent, making both fragments potentially important pieces of evidence. Extensive examination revealed that both stainless steel fragments had physical and chemical signatures consistent with the umbilical slide cover on the tail section of a Buk SA-11 missile. This slide cover protects the umbilical plug during missile storage and transport.

**Figure 11.**
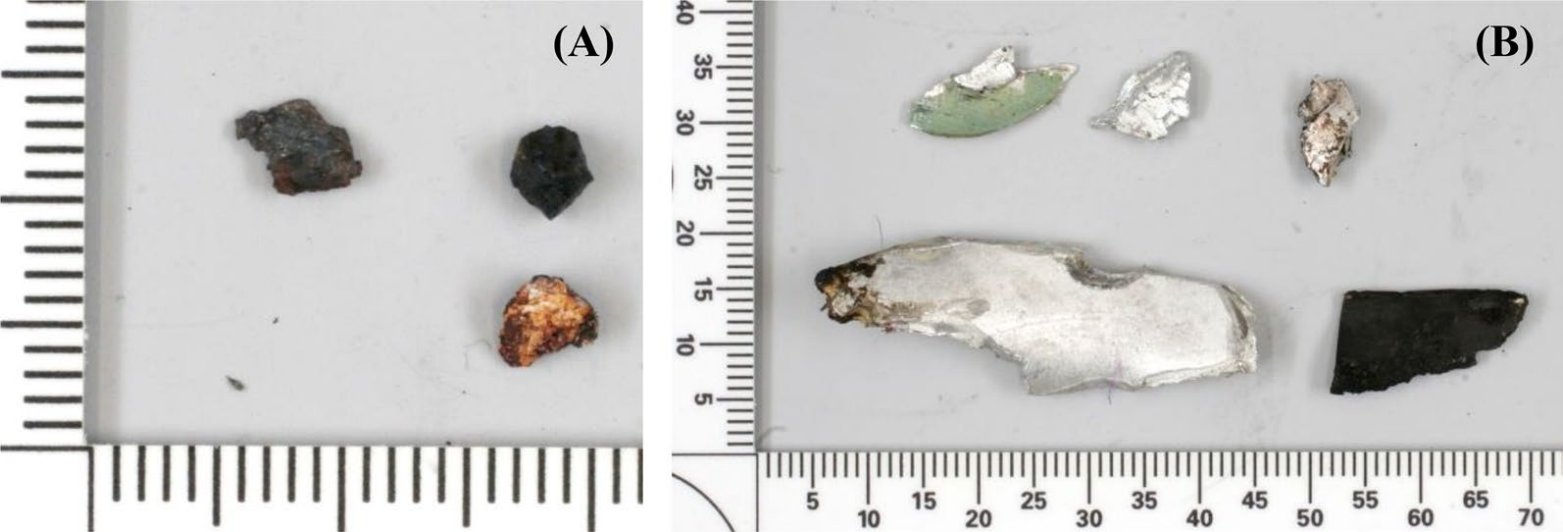
Photographs of fragments recovered from the remains of two passengers. (A) Three magnetic fragments recovered from a business-class passenger. (B) Five aluminium fragments recovered from an economy-class passenger. Scale: mm.

Nothing of forensic interest was found among the remains from passengers and other crew members. Most of the foreign objects were identified as personal belongings (e.g. rings, coins, telephones, zippers), objects originating from the airplane (seat belts, seat parts, airframe parts), or objects from the ground (e.g. stones, coal slag particles). Figure 11A shows three magnetic fragments recovered from the human remains of a business-class passenger. These fragments were initially thought to be steel but, after a thorough examination, were found to be coal slag. The crash site of MH17 is a mining area in which many roads are paved with coal slag. This coal slag is the likely source of the fragments in Figure 11A.

Many of the recovered objects consisted of aluminium-like fragments. Figure 11B shows five fragments of aluminium recovered from the human remains of an economy-class passenger. Boeing 777 aircraft and Buk missiles are constructed using different types of aluminium, which can be distinguished based on their microstructure and chemical composition. However, the composition of Buk missiles only became apparent when the first two were dismantled in November 2014. Therefore, during the examination of fragments recovered from human remains in Hilversum, little attention was paid to those that were aluminium-like because most were expected to originate from the airplane.

## Conclusion

During the forensic triage hundreds of foreign objects were recovered from the human remains. Among these fragments, about thirty were found that provided vital clues to the cause of the downing of Malaysian Airline flight 17 in Ukraine on 17 July 2014.

With the help of the results from the triage, within a few weeks early suspicions that MH17 was downed by a BUK-missile were supported. The airplane was hit on the front side by high energy steel fragments with shapes that were consistent with shapes of fragments from a 9N314M warhead, typically used in Buk SA-11 Missiles. The thin deposits of cockpit glass and aluminium on the recovered steel fragments indicate that the fragments were coming from outside the airplane. If the fragments were coming from an improvised explosive device within the airplane, the deposits of cockpit glass on fragments recovered from human remains would be inexplicable. The other postulated scenarios were also not supported. In particular, no materials characteristic of 30 mm anti-aircraft rounds or air-to-air missiles were found, indicating MH17 was not shot down by a Sukhoi SU-25 jet.

Finally, the DSB and the JIT concluded that flight MH17 was struck by a Buk SA-11 surface-to-air missile launched from pro-Russian separatist-controlled territory in Ukraine. According to the JIT, the Buk missile used originated from the 53rd Anti-Aircraft Missile Brigade of the Russian Federation and had been transported from Russia on the day of the crash, fired from a field in a rebel-controlled area, and the launcher returned to Russia after it was used to shoot down MH17 [19].

## Authors’ contributions

Erwin Vermeij participated in the triage and examination, evaluated the results and drafted the manuscript. Peter Zoon participated in the triage and examination, evaluated the results and reviewed the manuscript. Reza Gerretsen participated in the triage and reviewed the manuscript. Vincent Otieno-Alego participated in the examination and edited the manuscript. All authors contributed to the final text and approved it.

## References

[1] International Civil Aviation Organization. Annex 13. Aircraft accident and incident investigation. International Standards and Recommended Practices. 10th ed. Quebec, Canada, July 2010.

[2] Dutch Safety Board, Crash of Malaysian Airlines Flight MH17. The Hague, 22 October 2015. [cited 2021 Nov 10]. Available from: https://www.onderzoeksraad.nl/en/page/3546/crash-mh17-17-july-2014

[3] Barreveld DJ, editor. Air crash investigations — Shot down over Ukraine? — The crash of Malaysia Airlines Flight MH17. Lulu.com, 2019.

[4] Khoo LS, Hasmi AH, Abdul Ghani Aziz SA, et al. MH17: the Malaysian experience. Malays J Pathol. 2016;38:1–10. 27126658

[5] Ranson D. The loss of Malaysian Airlines Flight MH17: a forensic and humanitarian task. J Law Med. 2015;22:745–750. 26349375

[6] Bushberg JT, Seibert JA, Leidholdt EM, et al. The essential physics of medical imaging. Philadelphia (PA): Lippincott Williams & Wilkins, 2012.

[7] Hofman P, Alminyah A, Apostol M, et al. Use of post-mortem computed tomography in disaster victim identification. Updated positional statement of the members of the disaster victim identification working group of the International Society of Forensic Radiology and Imaging. J Rad Imag. 2019;19:100346.

[8] Rutty GN, Biggs MJP, Brough A, et al. Remote post-mortem radiology reporting in disaster victim identification: experience gained in the 2017 Grenfell Tower disaster. Int J Legal Med. 2020;134:637–643. 31250083 10.1007/s00414-019-02109-x PMC7044252

[9] de Jong L, Legrand L, Delabarde T, et al. Experience with postmortem computed tomography in the forensic analysis of the November 2015 Paris attacks. Forensic Sci Res. 2020;5:242–247. 33209509 10.1080/20961790.2020.1802686 PMC7646581

[10] Beauthier F, Van de Voorde W, Lefevre P, et al. Belgium experience in disaster victim identification applied in handling terrorist attack at Brussels Airport 2016. Forensic Sci Res. 2020;5:223–231. 33209506 10.1080/20961790.2020.1775932 PMC7646600

[11] Tracqui A, Deguette C, Delabarde T, et al. An overview of forensic operations performed following the terrorist attacks on November 13, 2015, in Paris. Forensic Sci Res. 2020;5:202–207. 33209503 10.1080/20961790.2020.1811487 PMC7646576

[12] Blum A, Kolopp M, Teixeira PG, et al. Synergistic role of newer techniques for forensic and postmortem CT examinations. AJR Am J Roentgenol. 2018;211:3–10. 29708781 10.2214/AJR.17.19046

[13] Ruder TD, Thali Y, Bolliger SA, et al. Material differentiation in forensic radiology with single-source dual-energy computed tomography. Forensic Sci Med Pathol. 2013;9:163–169. 23264199 10.1007/s12024-012-9398-y

[14] Winklhofer S, Stolzmann P, Meier A, et al. Added value of dual-energy computed tomography versus single energy computed tomography in assessing ferromagnetic properties of ballistic projectiles. Investig Radiol. 2014;49:431–437. 24566289 10.1097/RLI.0000000000000032

[15] Paulis LE, Kroll J, Heijnens L, et. al. Is CT bulletproof? On the use of CT for characterization of bullets in forensic radiology. Int J Legal Med. 2019;133:1869–1877. 30911839 10.1007/s00414-019-02033-0 PMC6811383

[16] Thornby J, Landheer D, Williams T, et al. Inconsistency in 9 mm bullets: correlation of jacket thickness to post-impact geometry measured with non-destructive X-ray computed tomography. Forensic Sci Int. 2014;234:111–119. 24378310 10.1016/j.forsciint.2013.11.002

[17] D’Arcy G, Márquez-Grant N, Lane DW. Baggage scanners and their use as an imaging resource in mass fatality incidents. Int J Legal Med. 2020;134:1419–1429. 31396702 10.1007/s00414-019-02132-y PMC7295821

[18] Beveridge A, editor. Forensic investigation of explosions. Boca Raton, FL: CRC Press, 2012.

[19] Netherlands Public Prosecution Service, Closing speech MH17 case. [cited 2022 Jan 4]. Available from: https://www.prosecutionservice.nl/topics/mh17-plane-crash/prosecution-and-trial/closing-speech-public-prosecution-service-december-2021

